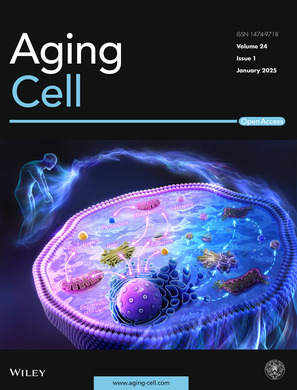# Featured Cover

**DOI:** 10.1111/acel.14478

**Published:** 2025-01-08

**Authors:** Yu Li, Jinxin Qi, Linhong Guo, Xian Jiang, Gu He

## Abstract

Cover legend: The cover image is based on the article *Organellar quality control crosstalk in aging‐related disease: Innovation to pave the way* by Gu He et al., https://doi.org/10.1111/acel.14447.